# Home- and community-based growth monitoring to reduce early life growth faltering: an open-label, cluster-randomized controlled trial

**DOI:** 10.3945/ajcn.117.157545

**Published:** 2017-08-23

**Authors:** Günther Fink, Rachel Levenson, Sarah Tembo, Peter C Rockers

**Affiliations:** 1 Harvard T.H. Chan School of Public Health, Boston, MA;; 2 Swiss TPH, Basel, Switzerland;; 3 Harvard University, John F. Kennedy School of Government, Cambridge, MA;; 4 Innovations for Poverty Action Zambia, Lusaka, Zambia; and; 5 Boston University School of Public Health, Boston, MA

**Keywords:** growth faltering, stunting, growth monitoring, height, weight, malnutrition

## Abstract

**Background:** Despite the continued high prevalence of faltering growth, height monitoring remains limited in many low- and middle-income countries.

**Objective:** The objective of this study was to test whether providing parents with information on their child’s height can improve children’s height and developmental outcomes.

**Design:** Villages in Chipata District, Zambia (*n* = 127), were randomly assigned with equal probability to 1 of 3 groups: home-based growth monitoring (HBGM), community-based growth monitoring including nutritional supplementation for children with stunted growth (CBGM+NS), and control. Primary study outcomes were individual height-for-age *z* score (HAZ) and overall child development assessed with the International Fetal and Newborn Growth Consortium for the 21st Century Neurodevelopment Assessment tool. Secondary outcomes were weight-for-age *z* score (WAZ), protein consumption, breastfeeding, and general dietary diversity.

**Results:** We enrolled a total of 547 children with a median age of 13 mo at baseline. Estimated mean difference (β) in HAZ was 0.127 (95% CI: −0.107, 0.361) for HBGM and −0.152 (95% CI: −0.341, 0.036) for CBGM+NS. HBGM had no impact on child development [β: −0.017 (95% CI: −0.133, 0.098)]; CBGM+NS reduced overall child development scores by −0.118 SD (95% CI: −0.230, −0.006 SD). Both interventions had larger positive effects among children with stunted growth at baseline, with estimated interaction effects of 0.503 (95% CI: 0.160, 0.846) and 0.582 (95% CI: 0.134, 1.030) for CBGM+NS and HBGM, respectively. HBGM increased mean WAZ [β = 0.183 (95% CI: 0.037, 0.328)]. Both interventions improved parental reports of children’s protein intake.

**Conclusions:** The results from this trial suggest that growth monitoring has a limited effect on children’s height and development, despite improvements in self-reported feeding practices. HBGM had modest positive effects on children with stunted growth. Given its relatively low cost, this intervention may be a cost-effective tool for increasing parental efforts toward reducing children’s physical growth deficits. This trial was registered at clinicaltrials.gov as NCT02242539.

## INTRODUCTION

Research estimates that globally, 167 million children (25.6%) aged <5 y have stunted growth, with prevalence rates >40% in several sub-Saharan African and South Asian countries (1, 2). While linear growth and stunted growth are widely used at the regional or national level as indicators for the nutritional status of children aged <5 y, height information is rarely provided to parents and caregivers in many low-income countries where routine health checkups for children <5 y old primarily focus on weight assessments to track children’s physical growth (3).

Given that weight-for-age *z* score (WAZ) is a function of both height and body mass, identifying faltering growth is difficult based on weight records alone. Although parents can compare their children with other children of the same age, such comparisons are not likely to provide reliable information in communities where developmental delays are common. As a result, a large number of caregivers of children with stunted growth are likely to be relatively unaware of physical growth delays experienced by their children unless they are extreme (4), which makes caregiver-initiated efforts to prevent or remediate faltering growth rather unlikely.

At the global level, interest has been increasing in designing and evaluating programs and interventions that aim to increase parental efforts to support child health and nutrition (5). A growing number of national governments have tried to change parental behavior through financial incentives (6–8) and by educating parents on best practices for raising their children, including practices related to feeding and nutrition (5, 9). In general, neither approach provides much information for parents, implicitly assuming that parents either do not need to know about their children’s developmental status or that such knowledge would not change their behavior because of other constraints. Neither of these assumptions seems obvious.

The main objective of this study was to test whether parental behavior and child growth outcomes can be improved by providing caregivers with increased access to height information. We tested 2 interventions designed to increase parents’ awareness of their children’s growth trajectories: *1*) the distribution of growth charts, which allow parents to directly monitor children’s growth trajectories at home, and *2*) the implementation of quarterly community-based growth-monitoring meetings, which allow parents to get clinical assessments of their children’s height on a quarterly basis close to their homes. Community-based platforms for monitoring growth have been tried in low-income settings, including South Africa, although minimal evidence exists on their effectiveness (10, 11).

## METHODS

### Trial design

The study was designed as an open-label, 3-arm, cluster-randomized controlled trial. Both treatments involved specific activities directly performed with households, so that all participants were fully aware of their treatment assignment from the beginning of the study. As described in detail below, the open-label designation did not apply to interviewers, who were generally not aware of the treatment status of households.

### Participants and eligibility

The study was nested within a larger longitudinal study conducted in 175 rural settlements in Chipata District, Zambia. As part of the larger study, 3175 randomly selected rural farming households with <12 acres of land (officially classified as “small-scale farmers”) had been enrolled in a study assessing the impact of seasonal credit access on agricultural output (see https://www.socialscienceregistry.org/trials/130 for details on the larger study). The overwhelming majority (>90%) of farmers in Zambia fall into this category, and virtually all of them are classified as poor, with estimated daily incomes <$1.25/person.

For the study reported here, we focused on households participating in the larger study that had ≥1 child between 6 and 24 mo of age at the beginning of the growth monitoring trial in September 2014. In some villages, <3 study households had a child in the target age range. To reduce the cost of fieldwork, these villages were excluded from the growth monitoring trial.

### Settings and locations

The study was conducted in Chipata District, Zambia. This district is part of Zambia’s Eastern Province, which has traditionally been one of the poorer areas of the country, with an estimated 43% prevalence of stunted growth in 2013 (12). The 2011 National Food and Nutrition Strategic Plan for Zambia identified prevention of stunted growth in children <2 y old as its Strategic Direction number 1 and the early identification, treatment, and follow-up of severe acute malnutrition as Strategic Direction number 3 (13). The ultimate objective of the national strategy is to reduce stunted growth among children <2 y old from 45% to 30% nationally.

### Interventions

#### Home-based growth monitoring

To enable parents to measure their children’s height and monitor children’s physical growth at home, 3 pilot versions of a growth chart were locally developed and tested. All 3 poster prototypes showed the height distribution for the WHO reference population. Specifically, for each age, the poster showed whether the child was in the green [height-for-age *z* score (HAZ) > −1], yellow (−2 < HAZ < −1), or red (HAZ < −2) zone. This color coding was adapted from midupper arm circumference (MUAC) measurement tapes most parents reported to be familiar with in focus groups.

The first prototype illustrated growth trajectories through the use of a baobab tree; the second poster illustrated growth through the use of maize stalks, and the third version simply showed growth curves at the bottom and a series of happy children of different ages at the top of the chart. The large majority of group participants expressed clear and strong preferences for the poster with the happy and healthy children. For the final poster used in the trial, we added nutrition information in the local language at the top of the chart. The 3 key messages highlighted were the importance of feeding young children 4–5 times/d, even if the quantities are not very large; the importance of protein in children’s diets; and the use of roller meal (coarsely ground maize flour including the shells, which contain some protein) instead of the usual maize flour. The charts were designed to be installed against a wall inside a house, with 50 cm between the floor and the bottom of the chart, so that children standing (with support for younger children) in front of the chart can be measured relatively easily by the caregiver (**Supplemental Figure 1**). To encourage routine measurement, we marked 3-mo age intervals, starting at 9 mo and ending at 30 mo, and instructed our delivery team to fill in the dates when children reached these age milestones. These prefilled dates were meant as prompts for parents and were not enforced in any way by the project team, who did not visit households after posters were installed and instructions given to parents. Separate charts were designed for boys and for girls, showing sex-specific reference standards and gender-specific healthy children on the poster. While some of the youngest children would not be able to stand without parental support at baseline, all children were assumed to be able to use the poster within 3 mo of the baseline survey and poster installation.

#### Community-based growth monitoring with targeted nutritional supplements

We organized 3 community meetings in all selected villages over the study period: a first visit in October 2014, a second visit in January 2015, and a third visit in April 2015. All meetings were organized and run by the study staff, who received anthropometric assessment training from the Ministry of Health’s district nutrition officer. In all community meetings, the 3 key messages highlighted were the importance of feeding young children 4–5 times/d, even if the quantities are not very large; the importance of protein in children’s diet; and the use of roller meal (coarsely ground maize flour including the shells, which contain some protein) instead of the usual maize flour. Equipment used to measure height and weight was rented from the National Food and Nutrition Commission. To make these community meetings more attractive to parents and to allow our staff to directly support parents of children experiencing faltering growth, nutritional supplements were provided to all children aged <2 y who were classified during community meetings as having stunted growth. Specifically, all children aged <2 y with HAZ <−2 were given 2 kg Yummy Soy, a locally manufactured food supplement based on soybeans and maize that contains vitamin A, thiamin, riboflavin, niacin, folate, vitamin C, vitamin B-12, iron, calcium, and zinc (see **Supplemental Tables 1** and **2** for details). Micronutrient content was confirmed through 3 random samples that were reanalyzed in the United States. Parents were provided a spoon for accurate measurement of the Yummy Soy powder and instructed to mix in the supplements with other food given to children, which typically was maize (*nshima*) porridge. Similar supplements were not provided to the control group or the home-based growth monitoring (HBGM) group.

Children in the control group received only an interview visit at baseline and an interview visit at the end of the study; no other services or materials were provided.

### Outcomes

The primary outcomes of the trial were children’s HAZ and children's overall development at the study’s end. Height, weight, and MUAC data used for analysis were collected by trained staff members at both baseline (September and October 2014) and the end of the study (July to September 2015). Height of children aged <2 y was measured with the child in a supine position; height for older children was measured with the child standing. Age- and sex-standardized *z* scores were computed for height, weight, and weight-for-height through the use of the WHO growth reference tables (14). We used the International Fetal and Newborn Growth Consortium for the 21st Century Neurodevelopment Assessment to assess child development at the end of the study (15). This tool was developed for the International Fetal and Newborn Growth Consortium for the 21st Century project to allow direct assessment of children aged ~2 y in culturally diverse high- and low-income settings (15).

To assess the impact of the intervention on parents’ behavior when feeding their children, a detailed food intake questionnaire was completed by all caregivers at the end of the study. We analyzed these as 4 separate outcomes: child’s consumption of roller meal, child’s consumption of standard flour, breastfeeding, and child’s consumption of proteins. For all four outcomes, parents reported consumption over the past week. We also analyzed consumption of Yummy Soy food supplements by children with stunted growth. Last, to test directly whether the intervention had an impact on parental behavior more generally and the relative priority given to child nutrition, we analyzed parental preferences for child nutrition relative to cash. As a thank-you for their survey participation, we gave parents the choice between a small cash gift or a small jar of peanut butter (of equal value) for their children. We then tested whether the interventions increased parental propensity to prefer nutritional food over cash.

Children in the HBGM and control groups were only visited at baseline and at the end of the study. Children in the group receiving community-based growth monitoring including nutritional supplementation (CBGM+NS) completed the same 2 interviews and assessments but were also invited to participate in 3 rounds of growth monitoring meetings in their communities.

### Sample size

The study was powered to detect a difference of 0.5 SD in HAZ and in normalized child development outcomes between any of the 3 groups, with power of 0.9 and an 0.05 α level, assuming a 2-sided test. Based on the latest Zambia Demographic and Health Survey (12), we anticipated a mean HAZ of −1.5 in the control group, with an intracluster correlation coefficient (ρ) of 0.2. Applying the design effect (DEFF) formula developed by Kish (16), this implied a DEFF of 2. With a DEFF of 2, 160 children/arm were required to achieve power of 0.9. We anticipated 20% attrition and thus aimed to enroll ∼200 children in each arm at baseline.

### Randomization and masking

Treatments were randomly assigned at the village level through the use of a computer-generated random draw by the principal investigator (GF); villages were stratified by parent study treatment (micro-credit program) and mean household size and harvest incomes reported in 2014. Blinding of subjects or assessors was not possible given the nature of the intervention.

### Implementation

Random draws for treatment assignment were generated in Stata software (StataCorp LLC) by the principal investigator. Growth charts were distributed immediately after the baseline survey; members of the study field team visited selected households and installed the posters themselves to ensure appropriate placement. No further home visits were made after the installation before the final assessment. CBGM+NS meetings resumed ∼2 wk after completion of the baseline survey. All baseline surveys were conducted between 25 September and 20 October 2014. All surveys at the end of the study were conducted between 8 July and 17 September 2015. Data were collected electronically with the use of handheld tablets; all data were managed by the study team.

### Statistical methods

Linear probability models were applied to test for differences in attrition rates across study arms. We used standard linear regression models to analyze continuous height and child development measures. We first estimated unadjusted mean differences and then estimated ordinary least squares models adjusting for a large range of baseline covariates, including HAZ and WAZ. To analyze stunted growth (HAZ <−2) and underweight (WAZ <−2), we used logistic regression models. We used linear probability models with heteroscedasticity-robust SEs to assess mean differences in parental behavior. For all models estimated, SEs were corrected for residual correlation at the cluster level through the use of the cluster-robust variance estimator developed by Huber (17). All models compare mean outcomes in the intervention group with mean outcomes in the control group at the end of the study.

Given that both interventions primarily focused on providing parents with information about children not reaching age-specific targets (e.g., HAZ <−2 showed children in the “red zone”), larger behavioral responses were likely for parents with children with stunted growth. To test this empirically, we performed subgroup analysis by children’s stunted growth status at baseline. Larger treatment effects also seemed likely for children aged <2 y, when most growth faltering seems to occur empirically (18). To test for such heterogeneous treatment effects, we estimated models stratified by age and formally tested for effect heterogeneity using fully interacted models. Given the limited power of the study and the low likelihood of finding large group differences over a 12-mo period with initially aligned outcome variables, no adjustments for multiple testing (multiplicity) were made.

### Human subjects

The study was approved by the Harvard T.H. Chan School of Public Health Office of Human Research Administration under protocol number IRB14-2948 and by ERES Converge in Zambia under reference no. 2014-June-011. Consent for study participation was obtained from the head of each household. Given the relatively low perceived risk of the study, no trial monitoring committee was set up for the study.

## RESULTS

**FIGURE 1:**
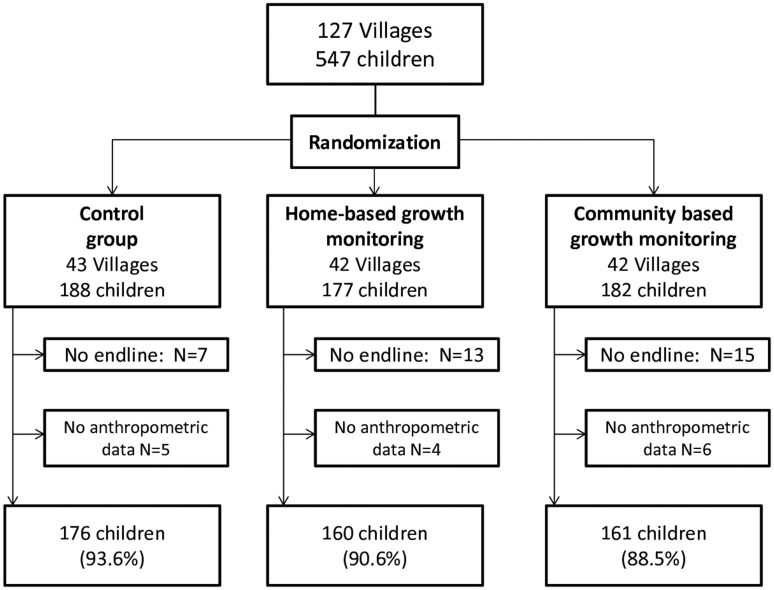
Enrollment and follow-up rates by study arm. A total of 547 children were enrolled at baseline. Of these, 497 children had complete outcome data and were used for these analyses.

**Figure 1** summarizes the overall study design and participant retention. A total of 547 of 569 targeted children (96.1%) and their caregivers were enrolled in the study across 127 villages in September and October 2014. We reassessed 512 children (93.6%) at the study’s end in September 2015. Of the 512 end-of-study surveys, 15 did not have valid anthropometric assessments: in 8 cases (1.5%) the caregiver refused measurement, and in 7 cases (1.4%) data were not recorded correctly on the mobile devices. No statistically significant differences were found in follow-up rates across groups (*P* = 0.70).

A total of 177 growth charts were installed by study staff in children’s homes in the HBGM group immediately after completion of the baseline survey. In the HBGM group, in most cases (530/547) only 1 child/household was in the study; in cases where 2 children from 1 household were in the study, 2 posters were given to the household. A total of 126 community meetings were held across the 42 CBGM+NS villages between October 2014 and May 2015. For these meetings, all children aged <5 y in the community were invited. A total of 3341 height assessments were made across the 3 rounds of meetings (see **Supplemental Table 3** for further information).

**Table 1** shows the baseline demographic and anthropometric characteristics for each group. On average, across the 3 groups, children were 14 mo old (median age: 13 mo) at baseline, with a mean HAZ of −1.3, a mean WAZ of −0.7, a mean weight-for-height *z* score of −0.06, and a mean MUAC of 14.8 cm. Despite the very low mean HAZ, no case of severe acute malnutrition (MUAC <11.5 cm) was encountered at baseline. Almost 90% of children were breastfed at baseline, and 1% was receiving antiretroviral therapy. Most caregivers had obtained primary education, and ∼20% of households were participating in a cash or maize loan program. A total of 71% of parents reported that their child was able to stand independently at baseline; the proportion of children reported as being able to stand alone increased from 5% at age 6 mo to 65% at 10 mo, 78% at 12 mo, and >95% at 15 mo.

**TABLE 1:** Baseline sociodemographic and anthropometric characteristics by group^1^

	Control	HBGM	CBGM+NS	*P* value (equal means test)
Age, mo	13.9 ± 5.1	13.9 ± 5.0	14 ± 5.0	0.976
Child is female	92 (50.8)	90 (54.9)	90 (53.9)	0.751
Child is a twin	4 (2.1)	8 (4.5)	2 (1.1)	0.281
HAZ	−1.4 ± 1.4	−1.3 ± 1.3	−1.3 ± 1.4	0.586
WAZ	−0.8 ± 1.1	−0.8 ± 1.0	−0.7 ± 1.0	0.581
WHZ	−0.1 ± 1.1	−0.2 ± 1.0	0 ± 1.3	0.326
MUAC	14.8 ± 1.3	14.9 ± 1.3	15.1 ± 1.4	0.163
Motor development	0 ± 1.0	0 ± 0.9	0 ± 1.0	0.831
Child is in fair health	39 (20.7)	29 (16.4)	27 (14.8)	0.360
Child is in poor health	27 (14.4)	19 (10.7)	25 (13.7)	0.553
Child is breastfed	166 (88.3)	160 (90.4)	162 (89)	0.767
Child is receiving ART	1 (0.5)	2 (1.1)	3 (1.6)	0.533
Caregiver education				
Primary	109 (58)	95 (53.7)	118 (64.8)	0.095
Secondary	31 (16.5)	41 (23.2)	39 (21.4)	0.201
Female head of household	22 (11.8)	22 (12.5)	33 (18.2)	0.255
Asset quintile				
2	39 (20.7)	43 (24.3)	34 (18.7)	0.433
3	44 (23.4)	32 (18.1)	44 (24.2)	0.337
4	32 (17)	36 (20.3)	34 (18.7)	0.697
5	36 (19.1)	32 (18.1)	37 (20.3)	0.861
Household member ages, y				
<5	1.6 ± 0.9	1.6 ± 1.2	1.5 ± 0.8	0.264
5–14	1.9 ± 1.6	2 ± 1.7	1.7 ± 1.5	0.343
15–64	2.6 ± 1.2	2.5 ± 1.2	2.7 ± 1.3	0.674
≥65	0.1 ± 0.3	0 ± 0.2	0.1 ± 0.2	0.217
Grandparents, *n*	0.5 ± 0.8	0.7 ± 0.8	0.7 ± 0.8	0.092
Participates in cash loan program	43 (23.6)	31 (18.3)	42 (23.7)	0.806
Participates in maize loan program	50 (27.5)	45 (26.6)	39 (22)	0.854

1 Data are mean ± SD or *n* (%). *P* values are based on a 3-sample mean comparison. Standard errors underlying *P* values estimated through the use of the cluster-robust variance estimator developed by Huber (17). ART, antiretroviral therapy; CBGM+NS, community-based growth monitoring including nutritional supplementation; HAZ, height-for-age *z* score; HBGM, home-based growth monitoring; MUAC, midupper arm circumference; WAZ, weight-for-age *z* score; WHZ, weight-for-height *z* score.

**Table 2** summarizes the program’s impact on primary and secondary outcomes. The top panel of Table 2 shows unadjusted estimates; the bottom panel shows the same models adjusted for the full set of baseline covariates listed in Table 1. Neither intervention had a statistically significant impact on HAZ overall, with estimated mean differences (β) in HAZ of 0.127 (95% CI: −0.107, 0.361) and −0.152 (95% CI: −0.341, 0.036) in the HBGM and CBGM+NS groups, respectively, at the end of the study. HBGM had no impact on child development overall [β: −0.017 (95% CI: −0.133, 0.098)], whereas CBGM+NS reduced overall child development scores by 0.118 SD (95% CI: −0.230, −0.006 SD).

**TABLE 2:** Intervention impact estimates^1^

	Mean difference				Odds ratio	
	HAZ	INTER-NDA	WAZ	Food diversity	Stunted growth	Underweight
Unadjusted						
HBGM	0.153 (−0.101, 0.407)	0.018 (−0.116, 0.153)	0.178^#^ (−0.021, 0.377)	0.159 (−0.543, 0.862)	0.794 (0.531, 1.189)	0.611 (0.300, 1.243)
CBGM+NS	−0.105 (−0.340, 0.131)	−0.050 (−0.182, 0.083)	0.018 (−0.178, 0.215)	−0.339 (−0.976, 0.297)	1.143 (0.769, 1.699)	0.804 (0.441, 1.467)
Control	Ref.	Ref.	Ref.	Ref.	Ref.	Ref.
Clusters, *n*	125	125	126	126	125	125
Observations, *n*	497	504	497	512	497	497
Adjusted						
HBGM	0.127 (−0.107, 0.361)	−0.017 (−0.133, 0.098)	0.183* (0.037, 0.328)	0.219 (−0.500, 0.938)	0.734 (0.407, 1.324)	0.725 (0.235, 2.234)
CBGM+NS	−0.152 (−0.341, 0.036)	−0.118* (−0.230, −0.006)	−0.066 (−0.189, 0.056)	−0.216 (−0.889, 0.457)	1.294 (0.689, 2.432)	1.091 (0.423, 2.816)
Control	Ref.	Ref.	Ref.	Ref.	Ref.	Ref.
Clusters, *n*	125	125	126	126	125	125
Observations, *n*	482	486	481	493	482	481

1 Results are from multivariate linear regression models. Coefficients displayed in columns 2–5 represent mean differences (95% CIs). Coefficients displayed in columns 6 and 7 represent ORs (95% CIs). All adjusted models include controls for age (months); sex; twin status; height, weight, and weight for height at baseline; health at baseline; caregiver education; household composition; and household wealth quintile. We used cluster-robust SEs to account for within-cluster correlation. We used *t* tests to test hypotheses. Stunted growth was defined as HAZ <−2. Underweight was defined as WAZ <−2. ^#^Significance at the 10% level. *Significance at the 5% level. CBGM+NS, community-based growth monitoring including nutritional supplementation; HAZ, height-for-age *z* score; HBGM, home-based growth monitoring; INTER-NDA, International Fetal and Newborn Growth Consortium for the 21st Century Neurodevelopment Assessment; Ref., reference category; WAZ, weight-for-age *z* score.

In terms of secondary outcomes, HBGM resulted in a mean WAZ increase of 0.183 (95% CI: 0.037, 0.328). No statistically significant differences were found for the other outcomes of interest in the HBGM group, although the point estimates suggest small positive impacts on development, food diversity, and stunting.

**Table 3** shows estimated interaction effects with baseline age and baseline stunted growth status. The impact of both interventions was more positive among children with stunted growth, with estimated interaction effects of 0.503 (95% CI: 0.160, 0.846) and 0.582 (95% CI: 0.134, 1.030) for CBGM+NS and HBGM, respectively. No interaction was found between baseline age and either intervention.

**TABLE 3:** Intervention interactions with baseline age and stunting status^1^

	HAZ		WAZ	
Outcome model	Unadjusted	Adjusted	Unadjusted	Adjusted
HBGM	−0.179 (−0.462, 0.105)	−0.042 (−0.283, 0.200)	0.038 (−0.199, 0.274)	0.135 (−0.078, 0.348)
HBGM × stunted growth at baseline	0.565* (0.085, 1.045)	0.582* (0.134, 1.030)	0.146 (−0.358, 0.650)	0.176 (−0.189, 0.541)
HBGM × age <12 mo at baseline	0.164 (−0.220, 0.548)	0.192 (−0.188, 0.573)	0.004 (−0.382, 0.389)	0.069 (−0.216, 0.354)
CBGM+NS	−0.442** (−0.746, −0.138)	−0.252* (−0.497, −0.007)	−0.246^#^ (−0.508, 0.016)	−0.160^#^ (−0.341, 0.022)
CBGM+NS × stunted growth at baseline	0.764** (0.251, 1.276)	0.503** (0.160, 0.846)	0.480* (0.050, 0.909)	0.169 (−0.147, 0.485)
CBGM+NS × age <12 mo at baseline	0.223 (−0.223, 0.670)	−0.093 (−0.459, 0.274)	0.280 (−0.107, 0.667)	0.056 (−0.251, 0.362)
Stunted growth at baseline	−1.550** (−1.878, −1.222)	−1.507 (−4.560, 1.546)	−1.069** (−1.341, −0.797)	−0.879 (−3.033, 1.276)
Age <12 mo at baseline	−0.311* (−0.601, −0.022)	−0.650 (−3.065, 1.764)	−0.228^#^ (−0.490, 0.033)	−1.772 (−3.910, 0.366)
Control	Ref.	Ref.	Ref.	Ref.
Clusters, *n*	126	125	126	125
*N*	497	482	497	481
*R*^2^	0.235	0.606	0.172	0.690

1 Results are from multivariate linear regression models. Coefficients represent mean differences (95% CIs). All hypotheses were tested with *t* tests. Adjusted models include all control variables displayed in Table 1 as well as interaction terms of baseline stunted growth and age <12 mo with all covariates. *Significance at the 5% level. **Significance at the 1% level. ^#^Significance at the 10% level. CBGM+NS, community-based growth monitoring including nutritional supplementation; HAZ, height-for-age *z* score; HBGM, home-based growth monitoring; Ref., reference category; WAZ, weight-for-age *z* score.

**Table 4** shows the estimated treatment effects on parental behavior. Both interventions seem to have triggered some changes in self-reported feeding behavior, with an additional 19.1% (95% CI: 0.090%, 0.291%) of caregivers in the HBGM group and an additional 13% (95% CI: 0.028%, 0.232%) in the CBGM+NS group reporting feeding their children coarsely ground maize including the kernels (roller meal), and a nonsignificant reduction in feeding children kernel-free maize flour. An additional 5.8% (95% CI: 0.001%, 0.114%) of families in the CBGM+NS reported use of food supplements. In terms of parental choices, HBGM increased by 14.7 percentage points (95% CI: 0.021, 0.274 points) the likelihood of parents opting for peanut butter rather than cash. A nonsignificant increase of 7.5 percentage points (95% CI: −0.058, 0.208 points) in the proportion of parents choosing peanut butter was found in the CBGM+NS group.

**TABLE 4:** Program impact on parental behavior^1^

	Child consumption in past 7 d					
	Maize kernels(“roller meal”)	Maize flour (“breakfast meal”)	Breast milk	Protein sources	Food supplements	Parent preferred peanut butter to cash
HBGM	0.191** (0.090, 0.291)	−0.064 (−0.151, 0.022)	0.045 (−0.031, 0.121)	0.114 (−0.390, 0.618)	0.001 (−0.034, 0.036)	0.147* (0.021, 0.274)
CBGM+NS	0.130* (0.028, 0.232)	−0.048 (−0.129, 0.034)	0.042 (−0.037, 0.121)	−0.259 (−0.677, 0.158)	0.058* (0.001, 0.114)	0.075 (−0.058, 0.208)
Control	Ref.	Ref.	Ref.	Ref.	Ref.	Ref.
Control group average/proportion^2^	0.13	0.89	0.31	5.01	0.02	0.29
Clusters, *n*	126	126	126	126	126	126
Observations, *n*	493	492	492	493	492	493
*R*^2^	0.092	0.043	0.495	0.074	0.060	0.089

1 Results are from multivariate linear regression models. Coefficients represent mean differences (95% CIs). All hypotheses were tested with *t* tests. **Significance at the 1% level. *Significance at the 5% level. All adjusted models include controls for age (months); sex; twin status; height, weight, and weight for height at baseline; health at baseline; caregiver education; household composition; and household wealth quintile. We used cluster-robust SEs to account for within-cluster correlation. CBGM+NS, community-based growth monitoring including nutritional supplementation; HBGM, home-based growth monitoring; Ref., reference category.

2 Average of outcome in control group: proportion of caregivers reporting food consumption by child (columns 1–5) or preferring peanut butter over cash gift (column 6).

## DISCUSSION

The results of the randomized controlled trial presented here suggest that interventions aiming to increase parents’ awareness of their children’s current height status have only limited impact on children’s physical growth and development overall. While neither intervention significantly affected the main outcomes of the trial, we observed positive changes for both weight and height in the HBGM group, although the height improvements were statistically significant only among children with stunted growth at baseline. Surprisingly, similar effects were not found for community-based growth monitoring, despite the fact that children with HAZ <−2 were provided with additional food supplements as part of these meetings. This finding does not seem to be driven by lack of parental interest in monitoring, as more than three-quarters of parents in the study, on average, attended the community meetings (Supplemental Table 3). Results from our interaction analysis suggest that the CBGM+NS intervention actually decreased mean HAZ and WAZ among children without stunted growth, whereas more positive effects were found for children targeted by nutritional supplements. While the relatively small sample size of the study limits our ability to identify directly the mechanisms underlying these results, it is possible that parents taking their children to community meetings incorrectly interpreted positive feedback received from official measurements (evidence of their children not having stunted growth) as a signal to not worry about their children’s nutritional status, and thus reduced their effort to support children. It is also possible that parents attending community measurement meetings were disappointed by the fact that their child was not eligible for the food supplements provided to children with stunted growth and thus became less concerned about their children’s nutritional status. Last, as with all trials, we cannot rule out the possibility that this finding is the result of measurement or sampling error.

In terms of self-reported behaviors, both intervention groups seem to have absorbed at least some of the key messages, with increased use of the more protein-rich coarsely ground maize flour and some small (statistically insignificant) increases in breastfeeding. Remarkably, however, the very basic poster intervention seems to have achieved larger impacts on all observed behaviors, despite the fact that community meetings were held by trained health workers who kept reminding caregivers of the principles and importance of healthy nutrition as part of each session. The behavioral changes observed for growth charts do not, however, seem to be the result of frequent poster use; when prompted at the end of the study, caregivers reported actively using the poster about once every 2 mo, on average, which implies a measurement frequency similar to that of the quarterly community meetings. On the other hand, 156 of 160 posters (97.5%) were still hanging at caregivers’ homes at the study’s end, with most caregivers reporting to be very pleased to have them. One of the main things caregivers liked about the final poster version tested was the explicit focus on children who will be successful in later life; while we do not have data to test this directly, it is possible that the overall design of the poster increased parents’ aspirations (19, 20) and their willingness to spend additional resources on their children’s nutrition, as evidenced by the small choice experiment conducted at the very end of the study. Further research is needed to better understand the mechanisms of this intervention and the general relation between parental information, parental aspirations, and growth in early life.

The trial presented has limitations. First, the overall sample size was relatively small. While we had initially powered the study to detect a relatively ambitious 0.5-SD improvement in HAZ, the final sample allowed us to detect somewhat smaller effects. The empirically observed DEFF in our study was only 1.1; together with the lower-than-anticipated attrition rate of 5%, the minimum detectable effect size in the final study sample was 0.39 SD, which is better than expected but still ambitious for relatively low-intensity interventions like the ones tested in this study.

Second, the study duration was relatively short, with only about 10 mo between baseline and the end of the study; larger treatment effects may be possible with longer follow-up periods, and more intensive interventions may also lead to larger impacts. Third, in terms of the final outcomes and parental behaviors reported, the timing of the study may not have been ideal. Zambia’s climate allows for only 1 agricultural cycle/y, with harvests starting in April and ending in July. Interviews at the end of this study were done shortly after the harvest and thus reflect parental behavior in times of a relatively abundant food supply. Larger behavioral differences may have emerged if the study had ended during the lean or hungry season, which typically occurs between January and March (21).

### Generalizability and external validity

The study setting chosen is representative of a large number of rural farming communities in South Asia and sub-Saharan Africa, with highly seasonal incomes and a majority of farming households living below international poverty thresholds. Given the high degree of heterogeneity in local nutrition and feeding customs, nonhomogeneous treatment effects seem likely. Further studies are needed to assess the general impact of the interventions tested.

### Interpretation

The results from this trial suggest that increasing parental knowledge does not induce large improvements in child growth outcomes overall. Given their relatively low cost, home-based growth charts may be a cost-effective tool to increase parental efforts toward reducing children’s physical growth deficits, particularly among children with stunted growth.
